# The Role of Navitoclax in Myelofibrosis

**DOI:** 10.7759/cureus.17976

**Published:** 2021-09-14

**Authors:** Sasirekha Pandravada, Steven Sandler

**Affiliations:** 1 Internal Medicine, Advocate Lutheran General Hospital, Park Ridge, USA; 2 Hematology/Oncology, NorthShore University HealthSystem, Skokie, USA

**Keywords:** navitoclax, myelofibrosis, bcl2 inhibitor, hematology, oncology, pmf, primary myelofibrosis

## Abstract

Primary myelofibrosis (PMF) is the most aggressive type of chronic myeloproliferative neoplasm, characterized by a disarray of hematopoietic stem cells and bone marrow fibrosis. The estimated incidence is 1.5 per 100,000 individuals per year with a median survival of less than six years. This statistic can vary by risk category, primarily based on clinical and cytogenetic features. Death can result from many causes, including leukemic transformation, cachexia, vascular events, and infection. Currently, allogeneic hematopoietic cell transplant is the only curative method for those at high risk. Unfortunately, only about 10% are eligible for this therapy. JAK2 kinase inhibitors are commonly used for high-risk patients with symptomatic splenomegaly or systemic symptoms from PMF. In clinical trials, the major endpoint is a reduction of spleen size by 35%. Secondary endpoints have included amelioration of symptomatic PMF and overall survival, which can be difficult to determine because of frequent co-morbid conditions. Current Food and Drug Administration (FDA)-approved JAK2 inhibitors have not shown increased survival or reduced risk of leukemic transformation. In relapsed or refractory disease, there is currently no standard of care. In this paper, we discuss the role of a new anti-apoptotic B cell leukemia 2 (Bcl-2) inhibitor, Navitoclax, for the treatment of myelofibrosis. The clinical data thus far for Navitoclax, especially in synergistic combination with traditional JAK2 inhibitors, have been promising for those with a refractory or relapsing disease on prior therapies. Following the encouraging results of phase II trials, ongoing phase III trials will primarily evaluate splenic size reduction versus the standard of care and evaluate secondary endpoints such as symptom reduction and overall survival. These studies may establish a new standard of care for refractory or relapsed myelofibrosis.

## Introduction and background

Navitoclax is a novel anti-apoptotic B cell leukemia 2 (Bcl-2) inhibitor that, when combined with ruxolitinib in phase II trials, has shown significant promise in the treatment of intermediate-2 to high-risk myelofibrosis [1]. Primary myelofibrosis (PMF) is one of the chronic myeloproliferative neoplasms (MPN). It is classified as a hematological disorder that is characterized by a clonal hematopoietic stem cell disorder resulting in chronic myeloproliferation, megakaryocytic hyperplasia, and excessive scarring of the bone marrow. The bone marrow fibrosis replaces normal hematopoiesis, which leads to extramedullary hematopoiesis and can ultimately evolve into acute leukemia [2]. The estimated incidence is 1.5 per 100,000 individuals per year, with the average age of diagnosis at 67 [3]. The features include hepatosplenomegaly, anemia which could be transfusion-dependent, and a peripheral smear with leukoerythroblastic features, along with other constitutional symptoms [2].

The diagnosis may be difficult to separate from other chronic myeloproliferative disorders, including chronic myeloid leukemia (CML), essential thrombocytosis, polycythemia vera, and MPN unclassifiable. The World Health Organization (WHO) subclassifies patients with PMF into prefibrotic PMF or overt PMF based on the degree of fibrosis [4]. The criteria needed for the diagnosis of PMF is made with the presence of major and minor criteria as defined by WHO in Table 1 [4-5].

**Table 1:** 2016 World Health Organization major and minor criteria for diagnosing PMF Table adapted from Barbui T et al. and Arber et al. [4-5] BM = bone marrow; CML = chronic myeloid leukemia; MDS = myelodysplastic syndrome; LDH = serum lactate dehydrogenase; WBC = serum white blood cell count; PMF = primary myelofibrosis a Diagnosis of early PMF requires all three major criteria and at least one minor criterion. Diagnosis of overt PMF requires all three major criteria and at least one minor criterion.

Primary Myelofibrosis (PMF)	
Prefibrotic/early PMF (pre-PMF)^a^	Overt PMF^a^
Major Criteria	Major Criteria
Megakaryocytic proliferation and atypia, without reticulin fibrosis > grade 1, accompanied by increased age-adjusted BM cellularity, granulocytic proliferation, and often decreased erythropoiesis	Megakaryocyte proliferation and atypia accompanied by other reticulin and/or collagen fibrosis (grade 2 or 3)
Not meeting WHO criteria for BCR-ABL + CML, PV, ET, MDS, or other myeloid neoplasm	Not meeting WHO criteria for BCR-ABL + CML, PV, ET, MDS, or other myeloid neoplasm
Presence of JAK2, CALR, or MPL mutation or in the absence of these mutations, presence of another clonal marker, or absence of minor reactive BM reticulin fibrosis	Presence of JAK2, CALR, or MPL mutation or in the absence of these mutations, presence of another clonal marker, or absence of minor reactive BM reticulin fibrosis
Minor Criteria	Minor Criteria
Presence of one or more of the following confirmed in two consecutive determinations: Anemia not attributed to a comorbid condition, WBC > 11 x 10^6/mL, palpable splenomegaly, LDH level above the upper limit of the institutional reference range	Presence of one or more of the following confirmed in two consecutive determinations: Anemia not attributed to a comorbid condition, WBC > 11 x 10^6/mL, palpable splenomegaly, LDH level above the upper limit of the institutional reference range, leukoerythroblastosis

The pathogenesis of PMF includes hematopoietic stem cell disarray in addition to bone marrow fibrosis. Commonly, patients with PMF were noted to have clonal karyotypic abnormalities in hematopoietic cells at diagnosis. Fifty to 65% of patients have a deletion in the chromosome of the retinoblastoma gene (13q-), 20q-, and partial trisomy 1q [6-7]. Gain of chromosome 9p, which bears the Janus kinase 2 (JAK2) gene, is found in about 50% of patients with PMF [8]. Unlike the disarray of hematopoietic stem cells, bone marrow fibrosis does not have these chromosomal abnormalities. This is thought to be due to multiple reasons. One explanation is that bone marrow fibrosis may be mediated by cytokines released from neoplastic megakaryocytes and other clonal hematopoietic cells [9]. Findings also note the role thrombopoietin (TPO) and its receptor, MPL, play in the pathogenesis of PMF in bone marrow fibrosis. Normal signaling involves TPO binding to the thrombopoietin receptor (MPL) and activating tyrosine kinase pathways, most notably the JAK/STAT pathway. The mechanism of fibrosis is thought to be from mutations in the TPO receptor, which is encoded by the c-MPL gene. Mutations in the MPL lead to increased JAK/STAT activation, leading to downstream effects of changes in cytokines and growth factors that promote fibrosis. Mutations in the calreticulin gene (CALR) have also been seen in patients with PMF promoting similar downstream activation of the JAK/STAT pathway [10-11]. Overall, 90% of patients with PMF have the JAK2, MPL, or CALR mutation, which are mutually exclusive [12].

Unfortunately, PMF is the most aggressive of the classic Philadelphia negative myeloproliferative neoplasms. The median survival is estimated to be less than six years with causes of death, including leukemic transformation, cachexia, vascular events, and infection [13].

The prognosis of PMF is associated with both clinical and genetic factors. Inferior survival was associated with clinical factors, including older age, anemia, leukocytosis, thrombocytopenia, need for transfusion, increased blast count, and degree of bone marrow fibrosis [14]. Genetic factors are generally divided into three risk categories based on genetic stratification, as seen in Table 2 [15].

**Table 2:** Risk stratification of PMF by genetic factors PMF = primary myelofibrosis

Risk Category	Genetic Abnormalities
Very High Risk	Single or multiple abnormalities of -7, i(17q), inv(3)/3q21, 12p-/12p11.2, 11q-/11q23, or other autosomal trisomies not including +8/+9 (eg, +21, +19)
Favorable	Normal karyotype or sole abnormalities of +9, 13q-, 20q-, chromosome 1 translocation/duplication, or sex chromosome abnormality including -Y
Unfavorable	All other abnormalities

Additionally, driver mutations, including JAK2, CALR, and MPL, cause the activation of the JAK/STAT pathway and play a major role in disease pathogenesis. Clinical outcomes appear to be more favorable for the CALR mutations and appear much less favorable for triple-negative (lack of JAK2, CALR, MPL) mutations [13]. Next-generation sequencing (NGS) is further defining the prognostic factors of PMF. High molecular risk (HMR) mutations, detected by NGS, are mutations of certain genes that have been associated with poorer prognosis. These include ASXL1, SRSF2, U2AF1Q157, EZH2, and IDH1/2 [16].

More recent prognostic models using both cytogenetic and molecular features in conjunction with clinical features are how PMF prognosis is assessed. The prognostic modeling in PMF began with the International Prognostic Scoring System (IPSS), which is the preferred scoring system created by the International Working Group for MPN Research and Treatment (IWG-MRT). This scoring system is used at the time of diagnosis and uses five independent predictors of inferior survival, including clinical features such as age>65, hemoglobin<10 g/dL, leukocyte count >25x10^9/L, circulating blasts >/=1%, and constitutional symptoms. The presence of 0, 1, 2, or >/=3 adverse features is correlated to low, intermediate-1, intermediate-2, and high-risk disease, respectively [17-19]. The IWG-MRT then developed a dynamic prognostic model (DIPSS). This uses the same variables as IPSS but can be used at any time during the disease course beyond the time of diagnosis. DIPSS assigns two adverse points for hemoglobin <10 g/dL and the new scoring system is determined as low (0 adverse points), intermediate-1 (1-2 points), intermediate-2 (3-4 points), and high (5-6 points) [20]. A prognostic scoring system developed in 2018 is now using the genetically inspired prognostic scoring system (GIPSS), and if needed, further evaluation by mutation-enhanced international prognostic scoring system plus karyotype, 70+, version 2.0 (MIPSS70+v2.0). GIPSS involves the karyotype, driver mutations, and HMR mutations, and categorizes a patient into low, intermediate, and high risk. For those that fall into an intermediate risk based on GIPSS, a MIPSS70+v2.0 score is done. This involves clinical features of the severity of anemia, karyotype, and mutations. This stratifies patients into either higher risk PMF or lower risk PMF [15,21]. Those who are considered higher risk PMF are evaluated to see if they are eligible for allogeneic hematopoietic cell transplants.

Allogeneic hematopoietic cell transplant is the only curative method for those at higher risk but is also associated with a higher risk of procedure-related complications, limiting those who may be eligible for this therapy [22-23]. In lower-risk PMF or higher-risk patients who are ineligible for allogeneic hematopoietic cell transplant, treatment is generally symptom-guided. Therapy options include ruxolitinib, fedratinib, or hydroxyurea [24]. In the clinical setting, the first line is typically hydroxyurea, however, more recently, ruxolitinib and fedratinib have been approved for first-line usage [25]. Ruxolitinib is a JAK2 inhibitor and has been shown to reduce spleen volume by 35% in 41.9% of patients with myelofibrosis [26]. Fedratinib is also a JAK2 kinase inhibitor and has also been shown to reduce splenomegaly and symptom burden in 33% of patients with myelofibrosis [27]. It has been indicated in the treatment of patients with intermediate-2 or high-risk primary or secondary (post-polycythemia vera or post-essential thrombocythemia) myelofibrosis [28]. These agents aid in symptomatic relief but have not been shown to lengthen survival or reduce the risk of leukemic transformation. In lower-risk asymptomatic patients, observation is preferred over symptom-guided treatment. There is no standard of care for relapsed or refractory disease and, generally, patients are encouraged to participate in clinical trials.

In this review, we focus on a novel orally bioavailable, antiapoptotic Bcl-2 inhibitor, navitoclax, the clinical rationale for its use in the treatment of myelofibrosis, and its outcomes in clinical trials with other therapeutic agents for relapsed or refractory cases of myelofibrosis.

## Review

Biology of Bcl-2 family proteins

**Figure 1:**
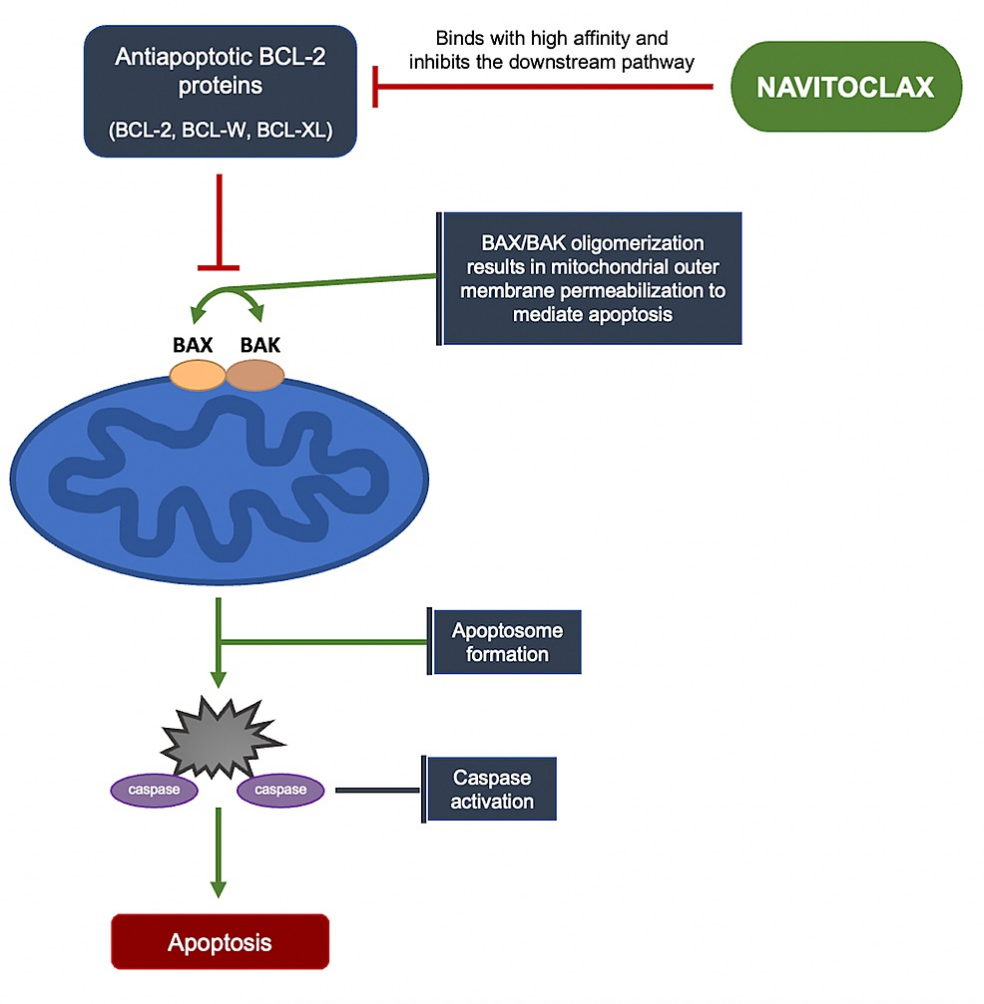
Mechanism of Action of Navitoclax Navitoclax binds with high affinity to anti-apoptotic proteins (Bcl-2, Bcl-W, Bcl-XL) and inhibits the downstream pathway of these proteins, causing BAX/BAK oligomerization, which permeates the mitochondrial outer membrane and activates caspase to ultimately cause apoptosis [30].

Apoptosis is regulated by prosurvival (anti-apoptotic) and proapoptotic proteins in the B cell leukemia 2 (Bcl-2) family. The anti-apoptotic proteins of the Bcl-2 family, such as Bcl-2 and Bcl-XL, prevent proapoptotic proteins that are needed to initiate mitochondrial apoptosis, which eventually leads to cancer cell survival. Navitoclax is a novel small molecule that targets and binds with high affinity to inhibit multiple anti-apoptotic Bcl-2 family proteins, including Bcl-XL, Bcl-2, and Bcl-W. As depicted in Figure 1, this, in turn, neutralizes the prosurvival proteins, causing BAX/BAK oligomerization, which are two nuclear-encoded proteins that pierce the mitochondrial outer membrane and activate caspases, which ultimately cause apoptosis [29-30]. In patients with myelofibrosis, the JAK2 mutation is associated with dysregulation of the Bcl-2 proteins [31]. Inhibition of Bcl-XL with Navitoclax induces significant apoptosis, preventing further cancer cell proliferation. In preclinical mice models, the Bcl-2 inhibitor, Navitoclax, in synergy with ruxolitinib-induced apoptosis to prevent cell proliferation and fibrosis. This combination was also thought to overcome acquired resistance to ruxolitinib [29,32].

Targeting Bcl-2 in myelofibrosis: Navitoclax

In phase II trials, Navitoclax combined with ruxolitinib functions in a synergistic manner to improve JAK2 inhibition. Patients with primary or secondary myelofibrosis who developed resistance to ruxolitinib in a first-line setting showed symptoms and spleen volume reduction with the addition of Navitoclax. In these trials, eligible patients were at least 18 years of age with a diagnosis of PMF or secondary (post-polycythemia vera or post-essential thrombocythemia) myelofibrosis who received pretreatment with ruxolitinib for at least 12 weeks prior to treatment initiation with Navitoclax. The protocol began with a 50 mg dose of Navitoclax daily with a combined stable dose of ruxolitinib of at least 10 mg twice daily. Weekly dose escalation of Navitoclax up to 300 mg was allowed, depending on tolerability and platelet count. The primary endpoint was spleen volume reduction percentage as determined by reduction from baseline MRIs. Secondary endpoints included total symptom score (TSS), overall response rate, rate of anemia response, improvement of bone marrow fibrosis, and safety profile [1]. The TSS consisted of 10 symptoms comprising fatigue, early satiety, abdominal discomfort, inactivity, problems with concentration, night sweats, pruritus, bone pain, fever, and unintentional weight loss, which were each rated 0 to 10. The bone marrow fibrosis was based on a grading system based on reticulin and collagen deposition in the bone marrow. In the phase II trials, of the 34 patients enrolled, half had primary myelofibrosis and the other half had secondary myelofibrosis. The median duration of pretreatment of prior ruxolitinib was 745 days. There were no triple-negative patients within the study. The results showed 29% of patients achieved >35% spleen volume reduction from baseline measured by MRIs. Furthermore, over 35% also achieved >50% TSS reduction. A reduction of driver mutation allelic burden of greater than 5% was noted in 42% of patients and bone marrow fibrosis was reduced by at least one grade in 25% of patients, which preliminarily suggests disease modification [1,33]. Finally, leukoreduction was also noted with 60% of patients, achieving transfusion independence. Thrombocytopenia was the most common adverse event, however, platelets eventually plateaued to 95x 10^9/L in six to eight weeks without any bleeding events. The degree and timing of thrombocytopenia is similar to that seen when using ruxolitinib alone [34]. There were no adverse event-related deaths [1,29]. Navitoclax demonstrated good tolerability in patients with a median duration of therapy of 330 days [33].

Currently, the ongoing phase III TRANSFORM-1 trial is evaluating the combination of Navitoclax and ruxolitinib vs placebo and ruxolitinib in adults with primary or secondary myelofibrosis who have not received JAK2 inhibitors. The ruxolitinib will be administered orally at a starting dose of 20 mg or 15 mg twice daily. This will be a double-blind, placebo-controlled study for those at least 18 years of age with intermediate-2 or high-risk MF with splenomegaly or MF-related symptoms and no prior treatment with JAK2 inhibitors. This study plans to include 230 participants enrolled in 130 sites worldwide [35]. A phase III TRANSFORM-2 trial is also ongoing, evaluating the combination of Navitoclax and ruxolitinib vs the best available therapy (BAT) in adults with relapsed or refractory myelofibrosis resistant to single-agent JAK2 inhibition. BAT options include hydroxyurea, interferon, ruxolitinib, fedratinib, or danazol, which will be administered at standard doses. The phase III study is designed to recruit those at least 18 years of age with intermediate-2 or high-risk myelofibrosis, splenomegaly, and myelofibrosis-related symptoms. Participants are to have had treatment with a prior JAK2 inhibitor for at least 24 weeks, which was stopped due to lack of efficacy or for less than 24 weeks with disease progression. The study is planned to be conducted in 173 sites in 23 countries with a sample size of 330 patients [36]. For both of these phase III trials, Navitoclax will be administered orally at a starting dose of 200 mg or 100 mg escalated to 200 mg if tolerated after >/= 7 days. The primary endpoint of the studies are at least a 35% reduction in spleen volume from baseline at Week 24 as measured by MRI or CT. The secondary endpoints include at least a 50% reduction in TSS from baseline at Week 24, duration of reduction in spleen volume from baseline, change in fatigue from baseline, time to deterioration of physical functioning, anemia response, reduction in grade of bone marrow fibrosis from baseline, overall survival, leukemia-free survival, and overall and composite response. Exploratory endpoints include progression-free survival. Safety is being monitored by adverse event monitoring, physical exams, vital sign measurements, lab testing, and electrocardiogram variables. Currently, these trials are in the recruiting stage [35-36].

In terms of future direction, the optimal therapy for refractory or relapsed myelofibrosis will need to be determined. Studies aimed to test different combinations of therapy or single agents are also avenues that need to be studied. Additional research is starting to evaluate newer clinical endpoints for myelofibrosis treatment. Currently, the clinical endpoints are a reduction in spleen volume and symptom burden. There are now studies that aim to look at factors such as progression-free survival, event-free survival, and overall survival. Other areas of research include determining which cytokines are upregulated and downregulated and how these are affected in the setting of JAK2 inhibitors and newer therapies such as Bcl-2 inhibitors. Determining the regulation of specific cytokines can also aid in drug development to prevent disease progression [33].

Until now, the combination of Navitoclax and JAK2 inhibitors has been promising. However, some limitations of Navitoclax include the dose-limiting thrombocytopenia that hindered its use in lymphoma treatment [37]. There are still questions about the long-term use of Navitoclax and its safety, tolerability, and duration of response. Future research into Navitoclax therapy, including optimal combination therapy, long-term outcomes, and the safety profile of the drug will need to be continually evaluated in patients with refractory or relapsed myelofibrosis.

## Conclusions

The addition of navitoclax to traditional JAK2 inhibitors in patients with myelofibrosis is demonstrating encouraging results in patients who are refractory or are relapsing on prior treatment therapy. This strategy based on early clinical trial data shows meaningful improvement in spleen volume reduction and total symptom score reduction. Patients with myelofibrosis in the intermediate-2 to high-risk groups have poor prognoses and are in need of therapeutic options. Based on the encouraging results of the phase II trial, the ongoing phase III trials may establish a new standard of care for refractory or relapsed myelofibrosis.
